# BRG1 overexpression in smooth muscle cells promotes the development of thoracic aortic dissection

**DOI:** 10.1186/1471-2261-14-144

**Published:** 2014-10-11

**Authors:** Yang Yuan, Chong Wang, Jibin Xu, Jin Tao, Zhiyun Xu, Shengdong Huang

**Affiliations:** Institute of Cardiothoracic Surgery, Changhai Hospital, Second Military Medical University, 168, Changhai Rd, Shanghai, P. R. China; Department of Cardiothoracic Surgery, Changhai Hospital, Second Military Medical University, 168, Changhai Rd., Shanghai, P. R. China

**Keywords:** Thoracic arotic dissection, Smooth muscle cells, BRG1, Apoptosis, Phenotype transition

## Abstract

**Background:**

Here we investigated Brahma-related gene 1 (BRG1) expression in aortic smooth muscle cells (SMCs) and its role in the regulation of the pathological changes in aortic SMCs of thoracic arotic dissection (TAD).

**Methods:**

BRG1, matrix metalloproteinase 2 (MMP2), and MMP9 mRNA and protein expression in human aortic specimens were examined by qPCR and western blot, respectively. The percentage of apoptotic and contractile SMCs in aortic specimens were determined by TUNEL assay and α-SMA immunohistochemical staining, respectively. The role of BRG1 in MMP2 and MMP9 expression, cell apoptosis, and phenotype transition in aortic SMCs were investigated using a human aortic SMC line via adenovirus mediated gene transfer. MMPs mRNA and protein levels were analyzed by qPCR and western blot, respectively. The percentage of apoptotic and contractile cells were determined through flow cytometry analysis.

**Results:**

The expression level of BRG1 in the aortic walls (adventitia-removed) was significantly higher in the TAD than the normal group. BRG1 expression was positively correlated to expression of MMP2 and MMP9 and SMC apoptosis, but was negatively correlated to the percentage of contractile aortic SMCs in TAD specimens. In human aortic SMC line, BRG1 transfection led to significant upregulation of MMP2 and MMP9 expression and a concomitant increase in SMC apoptosis as well as a decrease in the percentage of contractile phenotype of cells.

**Conclusions:**

BRG1 is significantly upregulated in the aortic SMCs of TAD, and its overexpression might promote the development of TAD by increasing MMP2 and MMP9 expression, inducing SMC apoptosis and the transition from contractile to synthetic phenotype.

## Background

Thoracic aortic dissection (TAD) is a life-threatening aortic catastrophe with high mortality [1–3]. It was reported that approximately 70% of untreated TAD cases would die during the first week following the occurrence of dissection [1–3]. The normal aortic media is mainly composed of concentrically arranged vascular smooth muscle cells (SMCs) and extracellular matrix (ECM) rich in elastic fibers, and the interplay between SMCs and ECM plays an important role for the structural and functional integrity of the aortic wall [4, 5]. Previous studies showed that overexpression of matrix metalloproteinase 2 (MMP2) and MMP9 in aortic SMCs might induce extracellular matrix degradation, apoptosis and phenotypic transition from the contractile to the synthetic type and that such pathological changes might result in instability of the aortic media, which are responsible for the occurrence of TAD [6–10]. It was reported that single gene mutation may cause aortic SMC dysfunction leading to the development of TAD [11, 12]. Over 80% of TAD cases, however, were found to be sporadic [5], suggesting that aortic SMC dysfunction may be mainly attributable to abnormal gene expression.

Brahma-related gene 1 (BRG1) is an ATPase subunit of the SWI/SNF complex, which is required for the transcriptional regulation of specific gene expression by chromatin remodeling [13, 14]. Previous studies showed that BRG1 plays an important role in the proliferation and differentiation of vascular cells [15–17]. Recently, it was found that BRG1 overexpression could induce cell apoptosis in rat mesenchymal stem cells and some human tumor cells [18–20]. Besides, it was reported that BRG1 could promote MMP2 and MMP9 expression in some human tumor cells [21, 22]. However, it is unclear whether BRG1 is involved in the regulation of MMP (especially MMP2 and MMP9) expression, apoptosis and phenotypic transition in aortic SMC during development of TAD.

In light of previous studies, we hypothesized that abnormal expression of BRG1 in aortic SMC might be involved in the development of TAD. To test the hypothesis, we investigated BRG1 expression in aortic SMCs of TAD and explored its role in the pathological processes of TAD. In clinical samples of TAD, BRG1 expression is significantly increased in aortic SMCs of TAD as compared with the controls. Moreover, the expression level of BRG1 was positively correlated with MMP2 and MMP9 expression and apoptosis index, but was negatively correlated with the proportion of contractile aortic SMCs in the aortic media of TAD. In a human aortic SMC line, we demonstrated that BRG1 overexpression markedly enhanced the expression of MMP2 and MMP9 and promoted apoptosis and the transition from the contractile to the synthetic phenotype. Our data suggest that BRG1 overexpression in aortic SMCs may be involved in the critical pathological processes that leads to the development of TAD.

## Methods

### Tissue collection

Aortic dissection specimens were obtained from 30 patients of acute type A TAD who underwent aortic replacement procedures at Changhai hospital in 2011. Aortic tissues from patients with hereditary connective tissue defects, such as Marfan’s syndrome, traumatic aneurysms or luetic aortic aneurysms were excluded. Control aortic specimens were obtained from 12 donors. The subjects with TADs and controls showed no significantly difference in clinical features including age, gender, smoking status, hypertension or diabetes. During surgery, full-thickness aortic wall specimens were collected from patients who underwent surgical repair of TAD (ascending aortas above the sinuses of Valsalva). Control specimens from normal ascending aortas were obtained from 12 organ donors. The aortic tissue was carefully cleared of adventitia, fixed in formalin for immunohistochemical staining or frozen fresh for Western blot analysis and qPCR.

The present study was conducted in accordance with the World Medical Association Declaration of Helsinki and was approved by the Medical Ethics Committee of Changhai Hospital. All participants signed informed consents.

### Cell culture

Human aortic SMCs (smooth muscle cells) line from healthy aortas was obtained from Cascade Biologics and was used up to passage 8. The cells were cultured according to supplier’s instructions, and were grown in Medium 231 with smooth muscle growth supplements (Cascade Biologics).

### Adenovirus mediated cell infection

Ad.BRG1 and Ad.Null were constructed using AdEasy system (Stratagene) according to user protocol. Human SMCs were infected with Ad.BRG1 or Ad.Null using a multiplicity of infection of 1:100 for 12 h at 37°C before further examination.

### Immunohistochemistry

Sections (5 μm) of the specimens were incubated with mouse anti-human primary antibodies (i.e. anti-BRG1, α-SMA, MMP2 and MMP9, Santa Cruz) overnight at 4°C, followed by incubation with horseradish peroxidase-conjugated goat anti-mouse antibody (Santa Cruz) for 1 hr at 37°C. Immunodetection was performed with the EnVision^TM^ Kit (Dako), using diaminobenzidine as the chromogen.

### TUNEL staining

The TUNEL reaction was performed with an in situ cell detection kit (Roche Applied Science, Indianapolis, IN, USA), according to the manufacturer’s instruction, to detect the apoptosis of media cells. TUNEL-positive nuclei (stained brown) were counted by Motic Images Advanced 3.2 in 10 random fields (×200), and then averaged.

### Apoptosis assay

Cell apoptosis was analyzed by annexin V-FITC assay. Briefly, cells were stained with annexin V-FITC and propidium iodide using the ANNEXIN V-FITC Kit (Beckman) according to the manufacturer’s protocol and subjected to flow cytometric analysis. Viable cells were not stained by annexin V or propidium iodide; early apoptotic cells were stained by annexin V but not propidium iodide whilst late apoptotic cells were stained by annexin V and propidium iodide.

### Protein extraction and Western blot analysis

For protein extraction, the tissue or cell samples were harvested in hypotonic lysis buffer (10 mM Tris–HCl, pH 7.5, 10 mM NaCl, 0.2 mM EDTA, 1 mM DTT) supplemented with inhibitors (25 mM b glycerol-phosphate, 25 mM NaF, 1 mM Na_3_VO_4_, 1 mM PMSF, 1 mM benzamidine,). Cell lysates were prepared by Dounce homogenisation and centrifuged at 500 g for 5 min to eliminate nuclei and debris. The supernatant was subjected to ultracentrifugation at 20,000 g for 60 min using the TLA-100.2 fixed angle rotor in Optima TL-100 ultracentrifuge (Beckman). The supernatant (cytoplasm) was adjusted to 100 mM NaCl and 0.5% Nonidet P-40. The membrane pellet was resolubilised in NETN buffer (50 mM Tris–HCl, pH 7.5, 100 mM NaCl, 200 mM EDTA, 0.5% Nonidet P-40, 1 mM DTT, supplemented with inhibitors).

Equal amounts of protein (50 μg) were separated by 10% SDS PAGE and then transferred to nitrocellulose membranes (NY, USA) by electroblotting. The membranes were blocked with 5% BSA in TBST (10 mM Tris–HCl, pH 8.0, 150 mM NaCl, and 0.05% Tween 20) for 1 hr, and then incubated with mouse anti-human primary antibodies at 4°C overnight before subsequent incubation with horseradish peroxidase-conjugated goat anti-mouse antibody (BD) for 1 hr at 37°C. Protein was visualized using enhanced chemiluminescence reagent (Santa Cruz). The expression level of target protein was analyzed using LabWork 4.0 program (UVP) and normalized to that of β-actin protein.

### Quantitative reverse transcription polymerase chain reaction (qRT-PCR)

Total RNA was extracted from 100 mg tissues or 1 × 10^5^ cells using the RNeasy RNA Mini Kit (Qiagen). First strand cDNA was synthesized using POWERSCRIPT reverse transcriptase (Clontech). The following gene-specific primer pairs were used for quantitative PCR:

*BRG1*: Forward, 5′- TCATGTTGGCGAGCTATTTCC -3′;

Reverse, 5′- GGTTCCGAAGTCTCAACGATG-3′.

*MMP2*: Forward, 5′- TTACTTGTGGAGCCGCTGAC -3′;

Reverse, 5′- TCAGATGGTGCCAGCAATAG -3′.

*MMP9*: Forward, 5′- GCTATTTCGGCATGTTGATCC -3′;

Reverse, 5′- GAAGTTAACCTCGGATCCTGG-3′.

*GAPDH*: Forward, 5′- GCTGAGTATGTCGTGGAGTC -3′;

Reverse, 5′- AGTTGGTGGTGCAGGATGC -3′.

PCR was performed using a Fast Start Master SYBR Green Kit (Roche) on a LightCycler (Roche). The expression level of target gene mRNA was analyzed using RealQuant software (Roche) and normalized to that of *GAPDH* mRNA.

### Statistical analysis

Statistical significance was tested using SPSS15.0 software. The correlation between the expressions of two genes was analyzed using Pearson’s correlation analysis. Other data are presented as mean ± SEM from 4 independent experiments, using student *t* tests for 2-group comparison. A *P* value less than 0.05 is considered as statistically significant.

## Results

### BRG1 expression is upregulated in aortic SMCs of TAD specimens

**Figure 1:**
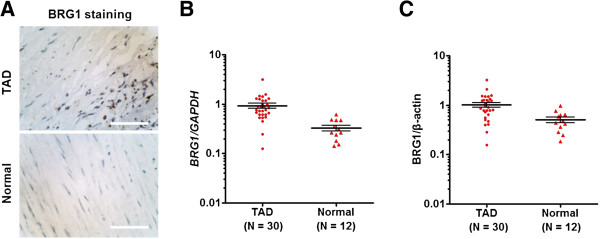
**BRG1 expression is upregulated in aortic SMCs of TAD specimens. (A)** Representative images of immunohistochemical staining for BRG1 in aortic media of TAD and normal aortic tissues. Expression level of BRG1 protein was moderate in TAD and was weak in normal tissue. Bar =50 μm. **(B, C)** The expression of BRG1 mRNA and protein in TAD and normal tissues was detected by qRT-PCR and western blot and normalized to that of *GAPDH* and β-actin, respectively. Each dot represents the relative expression level of BRG1 mRNA **(B)** and protein **(C)** of a tissue sample (n =30 for TAD, n =12 for normal tissues) with the line indicating the mean level; **, *P* <0.01 by paired *t* test.

In the present study, we first scanned the expression of BRG1 in TAD and normal aortic tissues by immunohistochemical analysis. It was found that BRG1 was mainly localized in the nucleus of aortic SMCs. Expression level of BRG1 protein was intense in TAD media, whereas it was low intense in the normal tissue. The number of BRG1 positive cells was significantly higher in TAD tissues than in normal aortic tissues (Figure 1A). Through qRT-PCR and western blot, we further revealed that the expression level of BRG1 mRNA and protein was 2.8 and 2.0 fold higher in the aortic media of TAD tissues compared with the normal tissues, respectively (Figure 1B and C). These data demonstrate that BRG1 expression is significantly upregulated in aortic SMCs of TAD specimens.

### BRG1 expression is positively correlated to the rate of apoptotic cells in aortic SMCs of TAD specimens

**Figure 2:**
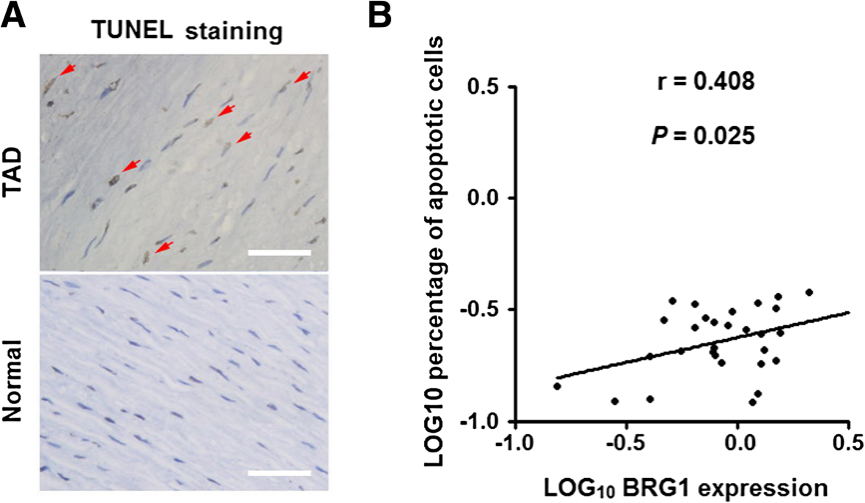
**BRG1 expression is positively correlated to the rate of apoptotic cells in aortic SMCs of TAD specimens. (A)** Representative images of TUNEL staining in aortic media of TAD and normal tissues. The percentage of TUNEL positive cells was significantly higher in TAD than in normal tissues. Bar =50 μm. **(B)** Dot plots represent log_10_percentage of apoptotic cells against log_10_BRG1 protein expression level. The lines represent approximated curves. The correlation coefficient (r) and the *P* value indicate the statistical significance of the positive correlation between the x and y variables.

Apoptosis is one of the mechanisms underlying aortic medial layer SMC loss. To investigate the effect of BRG1 on apoptosis of aortic SMCs, we analyzed the relationship between BRG1 expression and the apoptotic rate of aortic SMCs. As shown in Figure 2A, TUNEL-positive cells were hardly detectable in normal aortic tissues. In direct contrast, TUNEL-positive cells were numerous in the middle layer of TAD tissues. The number of TUNEL-positive cells in TAD tissues was significantly higher than that in the control group. Moreover, it was found that the expression of BRG1 was significantly positively correlated with the rate of TUNEL-positive cells in TAD tissues (Figure 2B).

### BRG1 expression is positively correlated to the expression of MMP2 and MMP9 in aortic SMCs of TAD specimens

**Figure 3:**
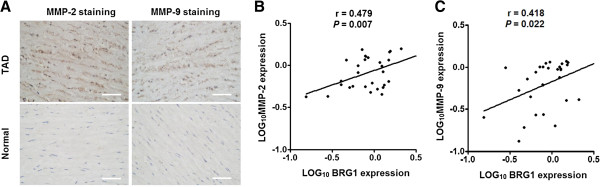
**BRG1 expression is positively correlated to the expression of MMP2 and MMP9 in aortic SMCs of TAD specimens. (A)** Representative images of immunohistochemical staining for MMP-2 and MMP-9 in aortic media of TAD and normal tissues. Expression level of MMP-2 and MMP-9 protein was significantly higher in TAD than in normal. Bars =50 μm. **(B, C)** Dot plots represent log_10_MMP-2 expression **(B)** and log_10_MMP-9 expression **(C)** against log_10_BRG1 protein expression level. The lines represent approximated curves. The correlation coefficient (r) and the *P* value indicate the statistical significance of the negative correlation between the x and y variables.

High expression of MMP2 and MMP9 was reported to contribute to the development of TAD. Consistent with the previous studies, immunohistochemistry assay showed that the expressions of MMP2 and MMP9 in TAD tissues were significantly higher than in the normal tissues (Figure 3A). To investigate the possible relationship between BRG1 and MMP2/9 expression, we performed correlation analysis of the western blot data and found that expression of BRG1 was significantly positively correlated with the expression of MMP2 (and MMP9) in TAD tissues (Figure 3B), suggesting that BRG1 might increase the expression of MMP2 and MMP9 in aortic SMCs of TAD specimens.

### BRG1 expression is negatively correlated to the rate of contractile SMCs in TAD specimens

**Figure 4:**
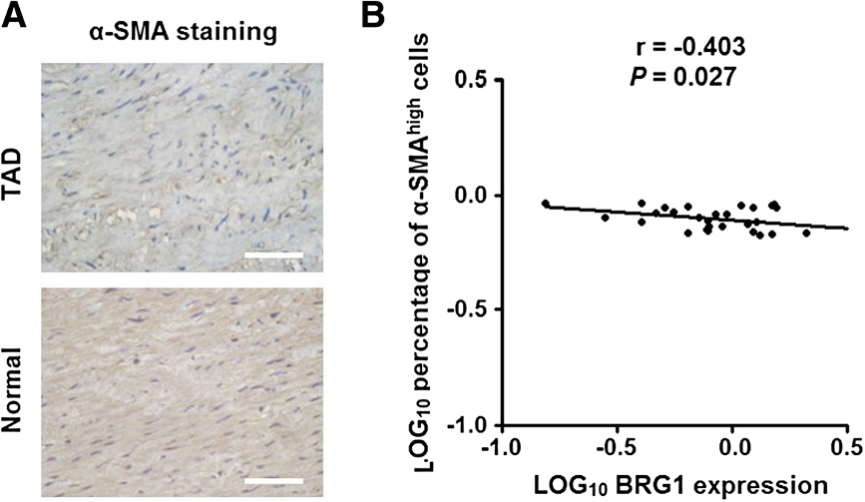
**BRG1 expression is negatively correlated to the rate of contractile SMCs in TAD specimens. (A)** Representative images of immunohistochemical staining for α-SMA in aortic media of TAD and normal tissues. The percentage of α-SMA positive cells in TAD tissues were significantly lower than that in the normal tissues. Bars =50 μm. **(B)** Dot plots represent log_10_percentage of α-SMA^high^ cells against log_10_BRG1 protein expression level. The lines represent approximated curves. The correlation coefficient (r) and the *P* value indicate the statistical significance of the negative correlation between the x and y variables.

Previous studies showed that aortic SMCs transited from contractile to synthetic phenotype cells led to reduce the contraction of aortic SMCs and thus weaken the aortic wall. α-SMA is widely used as a marker of the contractile phenotype of SMCs. Coinciding with the previous studies, immunohistochemistry assay showed that the expressions percentage of α-SMA positive cells in TAD tissues were significantly lower than that in the normal tissues (Figure 4A). Moreover, it was found that the expression of BRG1 was significantly negatively related to the percentage of α-SMA-positive cells in TAD tissues (Figure 4B). These data suggest that BRG1 might transit aortic SMCs from contractile to synthetic phenotype cells in TAD specimens.

### Overexpression of BRG1 induces apoptosis of aortic SMCs

**Figure 5:**
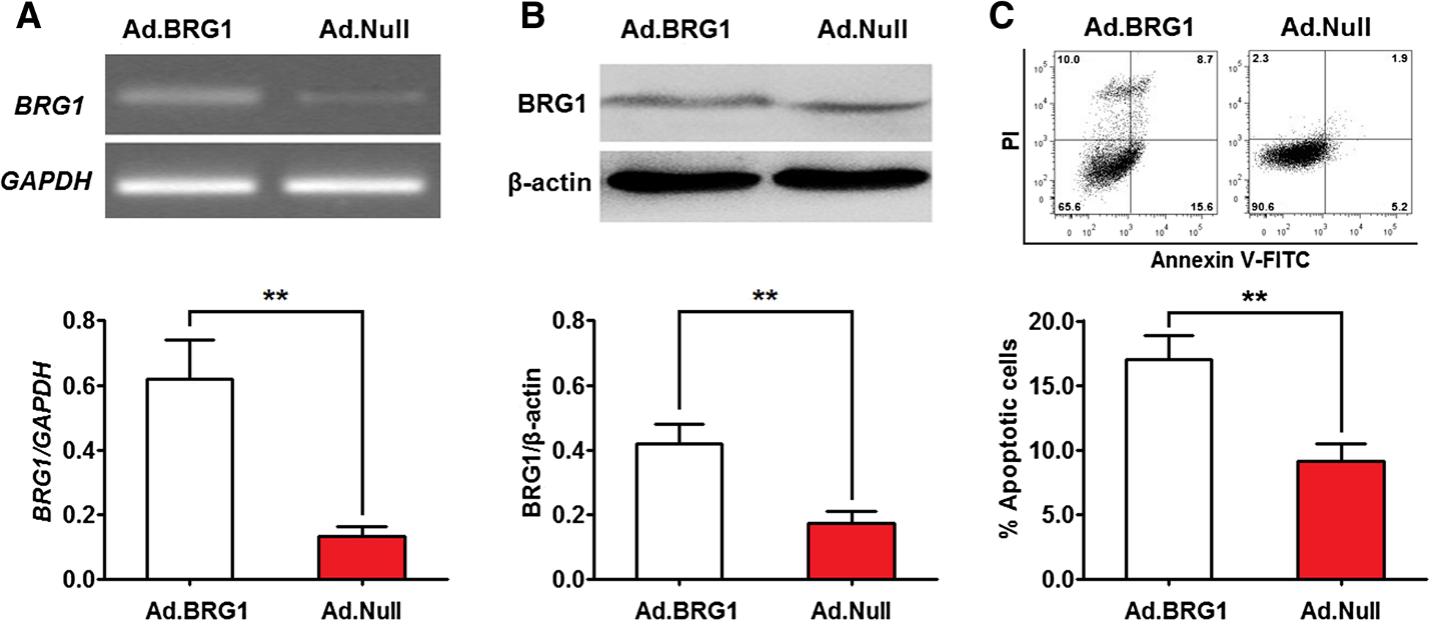
**Overexpression of BRG1 induces apoptosis of aortic SMCs.** Aortic SMCs were infected with Ad.BRG1 (or Ad.Null) at a MOI of 100. The expression of BRG1 and its effect on cell apoptosis were detected at 72 hr post-infection. **(A, B)** The expression level of BRG1 mRNA and protein was detected by qRT-PCR and western blot and normalized to that of GAPDH and β-actin, respectively. Histogram showed the expression level of BRG1 mRNA **(A)** and protein **(B)** in Ad.BRG1-infected and Ad.Null-infected aortic SMCs. **(C)** Cells were stained with annexin V-FITC and propidium iodide (PI). Flow cytometric contour plots showed the percentage of stained cells. Histogram showed the percentage of Annexin V^+^/PI^-^ and Annexin V^+^/PI^+^ cells of Ad.BRG1-infected and Ad.Null-infected aortic SMCs. Data represent mean ± SEM from 4 independent experiments; **, *P* <0.01 by *t* test.

To determine the effect of BRG1 on the apoptosis of human aortic SMCs, the apoptosis of human aortic SMCs transfected Ad.BRG1 was detected by flow cytometry. It was found that the expression of BRG1 mRNA and protein was significantly increased in the aortic SMCs treated with Ad.BRG1 as compared to the cells treated with Ad.Null at 72 hr post-infection (Figure 5A and B). Using annexin V-FITC assay, we found that in human aortic SMC line, the percentage of annexin V-FITC^+^/PI^-^ and annexin V-FITC^+^/PI^+^ in Ad.BRG1 infected cells were both significantly higher than those in Ad.Null infected cells (Figure 5C). These data indicate that overexpression of BRG1 promotes apoptosis of human aortic SMCs.

### Overexpression of BRG1 increases MMP2 and MMP9 expression in aortic SMCs

**Figure 6:**
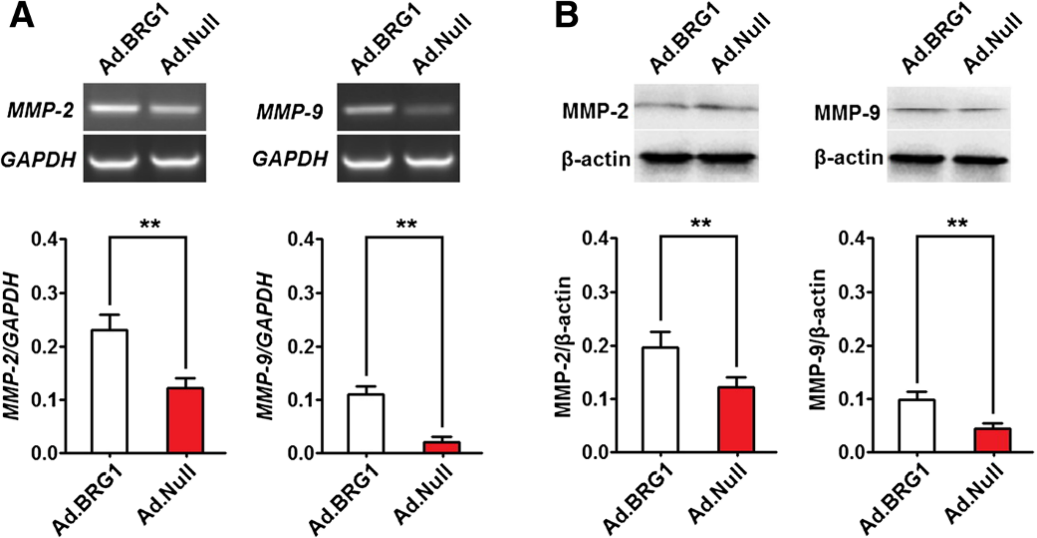
**Overexpression of BRG1 increases MMP2 and MMP9 expression in aortic SMCs.** Aortic SMCs were infected with Ad.BRG1 (or Ad.Null) at a MOI of 100. The expression of MMP2 and MMP9 were detected at 72 hr post-infection by qRT-PCR and western blot and normalized to that of GAPDH and β-actin, respectively. Histogram showed the expression level of MMP2 and MMP9 at the mRNA **(A)** and protein **(B)** level in Ad.BRG1-infected and Ad.Null-infected aortic SMCs. Data represent mean ± SEM from 4 independent experiments; **, *P* <0.01 by *t* test.

To investigate the effect of BRG1 on the MMP2 and MMP9 expression in aortic SMCs, MMP2 and MMP9 mRNA and protein levels in cultured human aortic SMCs treated with Ad.BRG1 were analyzed by qPCR and Western blotting. It was found that the expression of both MMP2 and MMP9 mRNA (or protein) in Ad.BRG1 infected cells was significantly higher than those in Ad.Null-infected cells (Figure 6A and B), suggesting that overexpression of BRG1 increases MMP2 and MMP9 expression in aortic SMCs.

### Overexpression of BRG1 promotes transistion of aortic SMCs from the contractile to the synthetic phenotype

**Figure 7:**
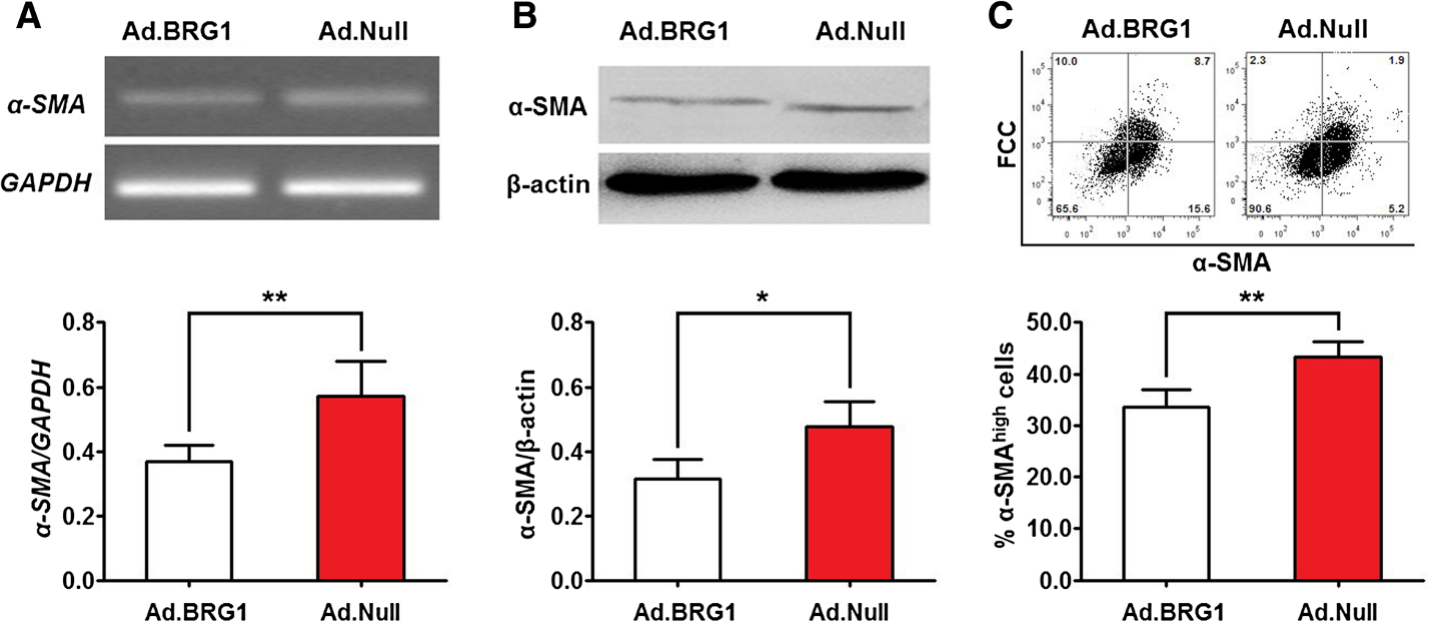
**Overexpression of BRG1 modulates aortic SMCs from contractile to synthetic phenotype.** Aortic SMCs were infected with Ad.BRG1 (or Ad.Null) at a MOI of 100. **(A, B)** The expression level of α-SMA mRNA and protein was detected at 72 hr post-infection.by qRT-PCR and western blot and normalized to that of GAPDH and β-actin, respectively. Histogram showed the expression level of α-SMA mRNA **(A)** and protein **(B)** in Ad.BRG1-infected and Ad.Null-infected aortic SMCs. **(C)** The percentage of α-SMA^high^ aortic SMCs were analyzed by flow cytometry. Flow cytometric contour plots showed the percentage of stained cells. Histogram showed the percentage of α-SMA^high^ cells of Ad.BRG1-infected and Ad.Null-infected aortic SMCs. Data represent mean ± SEM from 4 independent experiments; *, *P* <0.05 by *t* test.**, *P* <0.01 by *t* test.

To investigate the effect of BRG1 on the phenotypic transition of aortic SMCs, the expression of α-SMA was analyzed by qPCR and western blotting, and percentage of α-SMA-positive cells was analyzed by flow cytometry in cultured human aortic SMCs treated with Ad.BRG1. As shown in Figure 7A and B, the expression of α-SMA mRNA and protein was significantly lower in Ad.BRG1-infected cells than in Ad.Null-infected cells. In addition, the percentage of α-SMA-positively cell in Ad.BRG1 treated cells was significantly lower than that in Ad.Null treated cells (Figure 7C). These results suggest that overexpression of BRG1 may promote the transition of aortic SMCs from the contractile to the synthetic phenotype.

## Discussion

Mounting evidence supports the notion that thoracic aortic dissection (TAD) is the result of biomechanical weakening of the aortic media [1–3]. Previous studies revealed that matrix metalloproteinase 2 (MMP2) and MMP9 overexpression in SMCs might induce extracellular matrix degradation (especially the elastic fibers), apoptosis and phenotypic transition of SMCs (from contractile to synthetic types), and hence is strongly associated with the development of TAD [6–10]. Here, we show for the first time that BRG1 is overexpressed in the aortic SMCs of TAD, and that its overexpression can upregulate MMP2 and MMP9 expression, induce cell apoptosis and phenotypic transition in aortic SMCs. Our data suggest that BRG1 overexpression in aortic SMC contribute to the development of TAD.

It was reported that BRG1 is essential for the proliferation and differentiation in a variety of cell types including SMCs [15, 17, 23–26]. However, the role of BRG1 in TAD has not been investigated. In the present study, we first examine the expression of BRG1 in aortic walls using immunohistochemical staining. It was found that BRG1 was mainly localized at the nucleus of SMCs in the aortic media. Through qPCR and western blot analysis of the adventitia-free aortic walls, we further revealed that the expression of BRG1 in TAD specimens was significantly higher than in normal controls, suggesting that BRG1 might be involved in the development of TAD.

Extracellular matrix (ECM) degradation is wildly regarded as a key pathological process in TAD formation [6, 8]. Matrix metalloproteinase family is a group of protein enzymes responsible for maintaining the synthetic-lytic equilibrium of extracellular matrix [27, 28], amongst which MMP2 and MMP9 are members essential for ECM (especially elastic fibers) degradation [29, 30]. Ishii et al. showed that the expression level of MMP2 and MMP9 in SMCs of degenerated media was much higher than in other regions in TAD, suggesting that MMP2 and MMP9 overexpression in aortic SMCs is involved in TAD formation by promoting extracellular matrix degradation [31]. On the other hand, Ma et al. and Zhang et al. showed that BRG1 could induce MMP2 and MMP9 overexpression in some human tumor cells, respectively [15, 21]. In the present study, we demonstrated that the expression level of BRG1 was positively correlated with that of MMP2 and MMP9 in aortic media of TAD. In addition, using a human aortic SMC line, we show that BRG1 overexpression could significantly increase the mRNA and protein expression of MMP2 and MMP9, indicating BRG1 could promote MMP2 and MMP9 expression in SMCs probably at the transcriptional level. These results suggest that BRG1 overexpression may contribute to the development of TAD via upregulation of MMP2 and MMP9 expression in aortic SMCs and subsequent enzymatic degradation of the ECM.

Aortic SMCs are responsible for the tensile strength and elasticity of the aortic wall, which are the characteristic biomechanical properties of aorta representing its capacity to withstand stress [5, 11, 12]. Conceivably, loss of aortic SMCs would impair the mechanical strength of the vessel wall, leading to dilation and even rupture of the aortic wall [5]. It was reported that the number of apoptotic aortic SMCs in TAD was significantly higher than that in normal, suggesting that aortic SMCs apoptosis is involved in the formation of TAD [6, 7]. Previous studies showed that BRG1 overexpression could significantly increase the apoptotic rate of mesenchymal stem cells and some tumor cells [19, 20]. Recently, Zhang et al. showed that BRG1 was upregulated in vascular SMC of patients with primary atherosclerosis and was involved in the pathophysiological process of atherosclerosis presumably by inducing cell apoptosis [32]. In the present study, we found that BRG1 is overexpressed in aortic SMC and its level was positively correlated with the percentage of apoptotic aortic SMCs in the aortic media of TAD. Using an aortic SMC line, we further demonstrated that BRG1 overexpression can significantly induce aortic SMCs apoptosis. Our results suggested that BRG1 overexpression may promote the occurrence of TAD by inducing aortic SMCs apoptosis.

SMCs in the normal aorta are mostly quiescent cells that harbor a unique repertoire of contractile proteins required for cell functions [33]. It was reported that in response to vascular injury, the aortic SMCs could transit from contractile to a synthetic phenotype with little contractile proteins [34, 35]. Inamoto et al. and Huang et al. independently reported that the proportion of contractile aortic SMCs in the aortic media of TAD was significantly lower than in the normal aorta, suggesting that the transition of aortic SMCs from contractile to synthetic phenotype may play an important role in aortic SMCs contractile dysfunction in TAD [36, 37]. In the present study, we found that the level of BRG1 was positively correlated with the percentage of aortic SMCs with synthetic phenotype in TAD aortic media. In cellular experiment with an aortic SMC line, BRG1 overexpression was found to promote the transition from contractile to synthetic phenotype. The results imply that overexpression of BRG1 might be involved in the development of TAD by promoting phenotypic transition of aortic SMCs. It was reported that BRG1 could up-regulate the stemness genes such as Oct4, Sox2, Sall4, and maintain the self-renewal capacity in embryonic stem cells. As we known, the synthetic phenotype of aortic SMCs was regarded as the dedifferentiated cells. In this sense, it would be interesting to see whether BRG1 promote phenotypic transition of aortic SMCs through its regulation on the expression of stemness genes in the future.

However, our study had several limitations that should be mentioned. Low rate of proliferation is another important characteristic of contractile phenotype SMCs other than decreased production of MMPs, increased expression of SMC-specific contractile genes such as α-SMA and SM22α. Inflammatory cytokines such as IL-1β are another important factors regulating phenotype transition and induce expression of proinflammatory genes in SMCs. Whether BRG1 regulates SMC proliferation and the interaction between BRG1 expression and inflammatory states warrants further investigation. In addition, since the matrix degradation activity of the MMPs is also regulated by the presence of naturally present tissue inhibitors of metalloproteinases (TIMPs), examining the matrix degradation activity of the MMPs may better reflect the pathophysiologic role in the development and progression of the aortic disease than measuring MMP expression. Besides, interactions among MMPs expression, cell phenotypes transition and apoptosis would be investigated in the future. On the other hand, although our clinical investigation and in vitro cellular experiment suggest that BRG1 overexpression play roles in the development of TAD, further studies using appropriate animal models is still warranted.

## Conclusions

In summary, we report here that BRG1 level is significantly upregulated in aortic SMCs of TAD as compared with the controls in human samples, and its overexpression in human aortic SMC line can promote MMP2 and MMP9 expression, apoptosis and phenotypic transition from contractile to synthetic type. The results suggest that BRG1 overexpression in aortic SMC may play a role in the development of TAD. Further experiments in animal models are warranted to establish the role of BRG1 in the pathophysiological processes of TAD formation, and targeting BRG1 might provide a novel therapy in the treatment of TAD.

## Abbreviations

BRG1: Brahma-related gene 1
TAD: Thoracic aortic dissection
SMCs: Smooth muscle cells
MMP: Matrix metalloproteinase
ECM: Extracellular matrix.
